# Somatic cancer variant curation and harmonization through consensus minimum variant level data

**DOI:** 10.1186/s13073-016-0367-z

**Published:** 2016-11-04

**Authors:** Deborah I. Ritter, Sameek Roychowdhury, Angshumoy Roy, Shruti Rao, Melissa J. Landrum, Dmitriy Sonkin, Mamatha Shekar, Caleb F. Davis, Reece K. Hart, Christine Micheel, Meredith Weaver, Eliezer M. Van Allen, Donald W. Parsons, Howard L. McLeod, Michael S. Watson, Sharon E. Plon, Shashikant Kulkarni, Subha Madhavan, on behalf of the ClinGen Somatic Cancer Working Group

**Affiliations:** 1 Baylor College of Medicine and Texas Children’s Hospital, Houston, TX USA; 2 Ohio State University, Columbus, OH USA; 3 Innovation Center for Biomedical Informatics and Lombardi Comprehensive Cancer Center, Georgetown University Medical Center, Washington, DC USA; 4 National Center for Biotechnology Information, Bethesda, MD USA; 5 National Cancer Institute, Rockville, MD USA; 6 Illumina, San Diego, CA USA; 7 MolecularMatch, Houston, TX USA; 8 Invitae, San Francisco, CA USA; 9 Vanderbilt University School of Medicine, Nashville, TN USA; 10 American College of Medical Genetics and Genomics, Bethesda, MD USA; 11 Dana-Farber Cancer Institute, Boston, MA USA; 12 Moffitt Cancer Center, Tampa, FL USA

**Keywords:** Cancer genomics, Somatic variant interpretation, Data standard, Somatic variant curation

## Abstract

**Background:**

To truly achieve personalized medicine in oncology, it is critical to catalog and curate cancer sequence variants for their clinical relevance. The Somatic Working Group (WG) of the Clinical Genome Resource (ClinGen), in cooperation with ClinVar and multiple cancer variant curation stakeholders, has developed a consensus set of minimal variant level data (MVLD). MVLD is a framework of standardized data elements to curate cancer variants for clinical utility. With implementation of MVLD standards, and in a working partnership with ClinVar, we aim to streamline the somatic variant curation efforts in the community and reduce redundancy and time burden for the interpretation of cancer variants in clinical practice.

**Methods:**

We developed MVLD through a consensus approach by i) reviewing clinical actionability interpretations from institutions participating in the WG, ii) conducting extensive literature search of clinical somatic interpretation schemas, and iii) survey of cancer variant web portals. A forthcoming guideline on cancer variant interpretation, from the Association of Molecular Pathology (AMP), can be incorporated into MVLD.

**Results:**

Along with harmonizing standardized terminology for allele interpretive and descriptive fields that are collected by many databases, the MVLD includes unique fields for cancer variants such as Biomarker Class, Therapeutic Context and Effect. In addition, MVLD includes recommendations for controlled semantics and ontologies. The Somatic WG is collaborating with ClinVar to evaluate MVLD use for somatic variant submissions. ClinVar is an open and centralized repository where sequencing laboratories can report summary-level variant data with clinical significance, and ClinVar accepts cancer variant data.

**Conclusions:**

We expect the use of the MVLD to streamline clinical interpretation of cancer variants, enhance interoperability among multiple redundant curation efforts, and increase submission of somatic variants to ClinVar, all of which will enhance translation to clinical oncology practice.

**Electronic supplementary material:**

The online version of this article (doi:10.1186/s13073-016-0367-z) contains supplementary material, which is available to authorized users.

## Background

To achieve personalized medicine for oncology, it is critical to catalog and curate cancer genomic sequence alterations in order to improve biomarker applications, drug development, and treatment selection and ultimately reduce morbidity and mortality from cancer. Curation and interpretation efforts are underway in multiple centers, often through local internal repositories, web-based platforms curated by laboratory efforts [1], crowd-sourced cancer curation [2], or multi-institutional efforts such as Genomics, Evidence, Neoplasia, Information, Exchange (GENIE) [3]. However, there is no broadly adopted standardized framework to capture clinically relevant data on somatic variants. In order to streamline curation efforts of somatic alterations in the community and increase the clinical utility of cancer variant curation, the Somatic Working Group (WG) of ClinGen, with representation from groups including Clinical Sequencing Exploratory Research (CSER), Association of Medical Pathologists (AMP), American Society of Clinical Oncology (ASCO), and Global Alliance For Genomics and Health (GA4GH), in cooperation with ClinVar and multiple cancer variant curation stakeholders [1, 2, 4–6] has developed a consensus set of minimal variant level data (MVLD) that we propose for broad adoption as a standard framework to create a common language for curation and clinical interpretation of somatic alterations.

**Fig. 1:**
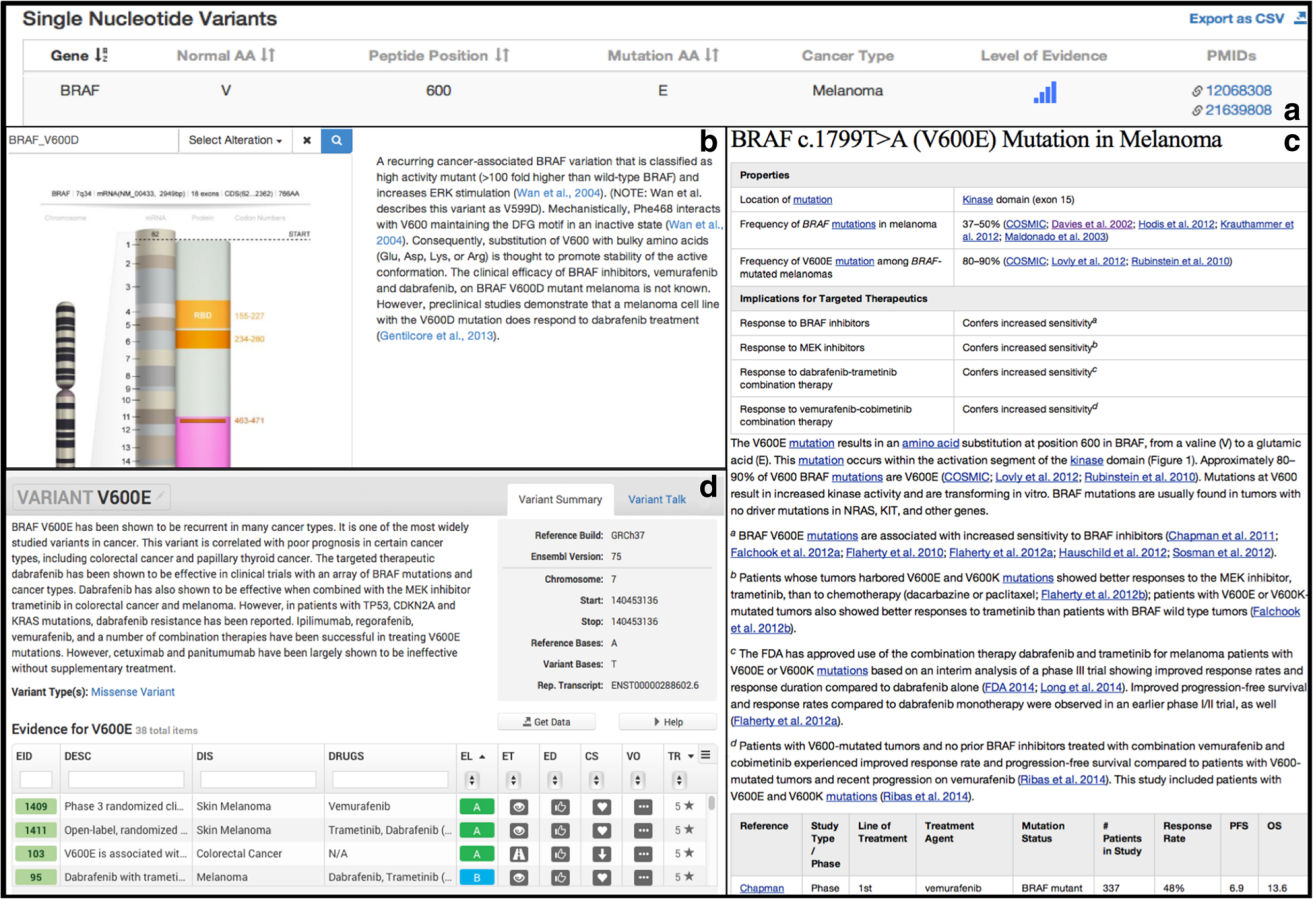
Selection of cancer variant interpretive databases. The images show the diversity of data collected, data formats, and displays for the somatic variants BRAF V600E/D in melanoma. **a** CanDL, **b** Personalized Cancer Therapy, **c** My Cancer Genome, **d** CIViC

The Clinical Genome Resource (ClinGen) is a National Institutes of Health (NIH) initiative representing over 75 institutions [6] and is a natural platform for supporting centralized curation of somatic variants and their interpretation. The ClinGen network focuses on clinical and public use of genetic information, with a special emphasis on curation of and education on gene-disease associations and variant interpretations. ClinGen has developed cancer-relevant Clinical Domain Working Groups, including Hereditary Cancer, Pharmacogenomics, and Somatic Cancer. Major challenges for current somatic variant curation include similar but inconsistent terminology, costly and redundant manual curation efforts, and lack of a central framework for housing and coordinating expertise. An example of the necessity of a common framework to describe somatic variants is shown in Fig. 1, where separate databases convey partial information about a somatic variant. Relevant elements are missing from all three; such discrepancies create information gaps and perceived inconsistencies and are a significant communication challenge for clinical or research use of variants.

## Methods

### Literature, institutional, and metadata reviews inform MVLD development

To develop MVLD, our team first reviewed clinical actionability interpretations from ten institutions participating in ClinGen Somatic WG, seeking information on guidelines or standards utilized to create their actionability frameworks and associated information. We also incorporated input from groups presenting frameworks on ClinGen Somatic WG conference calls [2, 7]. As guidance, we used prior effective efforts to define minimal and structured data, such as minimum information about a microarray experiment (MIAME) adopted by the Gene Expression Omnibus (GEO) database [8, 9]. In addition, we reviewed publications on clinical somatic variant interpretation [10–14]. We uncovered a wide variety of data elements but one distinct common theme emerged: many institutions (half from the survey and multiple others from literature review and group presentations) consistently promoted a somatic variant to the highest actionability if it had a corresponding Food and Drug Association (FDA)-approved drug therapy. The same institutions, as well as many others reviewed in the literature, had varying levels of lower priority tiers (between three to five tier schemes), with a general schema of incorporating lower priority levels of evidence such as guidelines from a professional society, on-going clinical trials, preclinical trials, case series, case reports, in vitro studies, or pathway-based evidence. Each group collected some part of the aforementioned information, and in a variety of formats and data collection systems. Using this as a rough guideline, we next reviewed fields that are currently in use from multiple somatic variant databases.

### Current systems capturing somatic variant data inform MVLD development

Databases with variant-level information relevant to cancer can be roughly divided into two types: variant catalogs and variant interpretive databases. Similar to the responding institutions and groups, each database captured somewhat analogous information, but often in distinct formats (Fig. 1). Variant catalogs include sites such as: Catalog of Somatic Mutations in Cancer (COSMIC) and International Cancer Genome Consortium (ICGC) data portal [15, 16]. Variant interpretive datasets include sites such as ClinVar, My Cancer Genome, Clinical Interpretations of Variations in Cancer (CIViC), the Cancer Driver Log (CanDL); multi-omics integration and analysis platforms include Georgetown Database of Cancer (G-DOC), cBIOPortal, and Personalized Cancer Therapy [1, 4, 5, 17–21]. Here, we briefly describe a selection of somatic variant interpretive sites reviewed in developing MVLD.

#### ClinVar

**Fig. 2:**
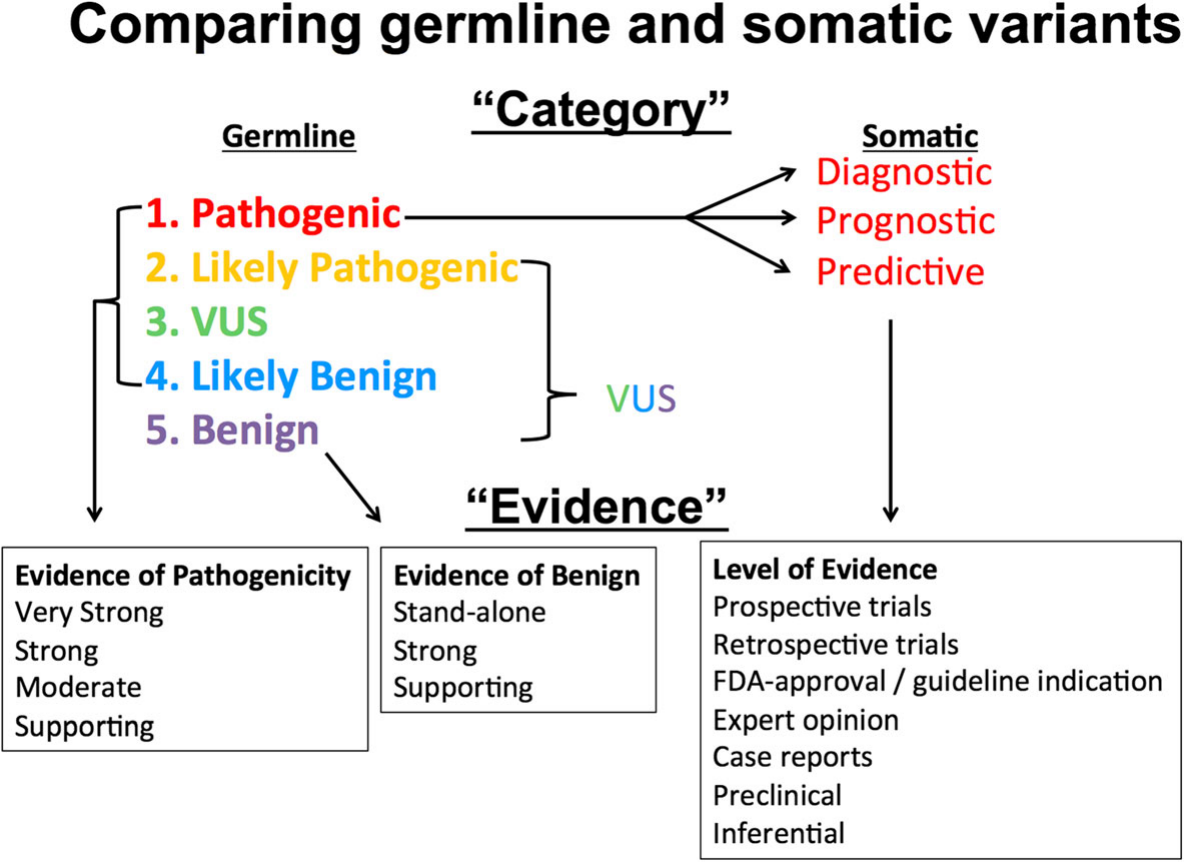
Comparison of germline and somatic variant categories and evidence. The *Pathogenic* category in germline is split into three categories for somatic: *Diagnostic*, *Prognostic*, and *Predictive*, *VUS* Variant of Unknown Significance

Of the cancer variant interpretive sites, ClinVar is the only database with a broader scope that extends beyond cancer to germline variants. ClinVar is an open, centralized variant repository of clinically relevant, interpreted variants and offers a summation of collected data as well as expert panel reviews. Currently, somatic alterations make up a minority usage of ClinVar, roughly 2 % of the total dataset. However, the infrastructure of ClinVar has the potential to be leveraged into somatic variant curation. For germline variant interpretation, there is a well-established approach [22]; a similar, though distinct, language is often applied for interpreting somatic variants (Fig. 2). For example, germline variants may be categorized as pathogenic, while somatic variants are often categorized as diagnostic, prognostic, or predictive biomarkers. Similarly, supporting evidence for a “very strong” pathogenic germline variant may come from published studies reporting high penetrance with segregation data, while supporting evidence for a predictive somatic variant could range from large randomized clinical trials to pre-clinical laboratory data. The Somatic WG worked closely with ClinVar in the development of MVLD, gaining insight into unique considerations in curating somatic variants from the perspective of experienced germline curators.

#### My Cancer Genome

My Cancer Genome was the first public somatic variant interpretation resource, launched in 2011, and includes information on the effect of tumor variants on sensitivity to targeted therapeutics, as well as a listing of cancer clinical trials that include biomarker information. The information is provided by expert contributors and edited by knowledge resource staff.

#### CIViC

CIViC provides descriptions and evidence levels for publications addressing tumor variants in cancer. The information is crowd-sourced and expert-moderated.

#### CanDL

CanDL is a curated list of cancer variants that have literature-defined levels of evidence for predicting response to therapy (predictive biomarkers only).

#### Personalized Cancer Therapy

Personalized Cancer Therapy is a password-protected knowledge resource with free and fee-based levels of access to the content containing information on the therapeutic implications of 27 genes in cancer, categorized by level of evidence.

## Results

### MVLD to describe cancer variants

**Fig. 3:**
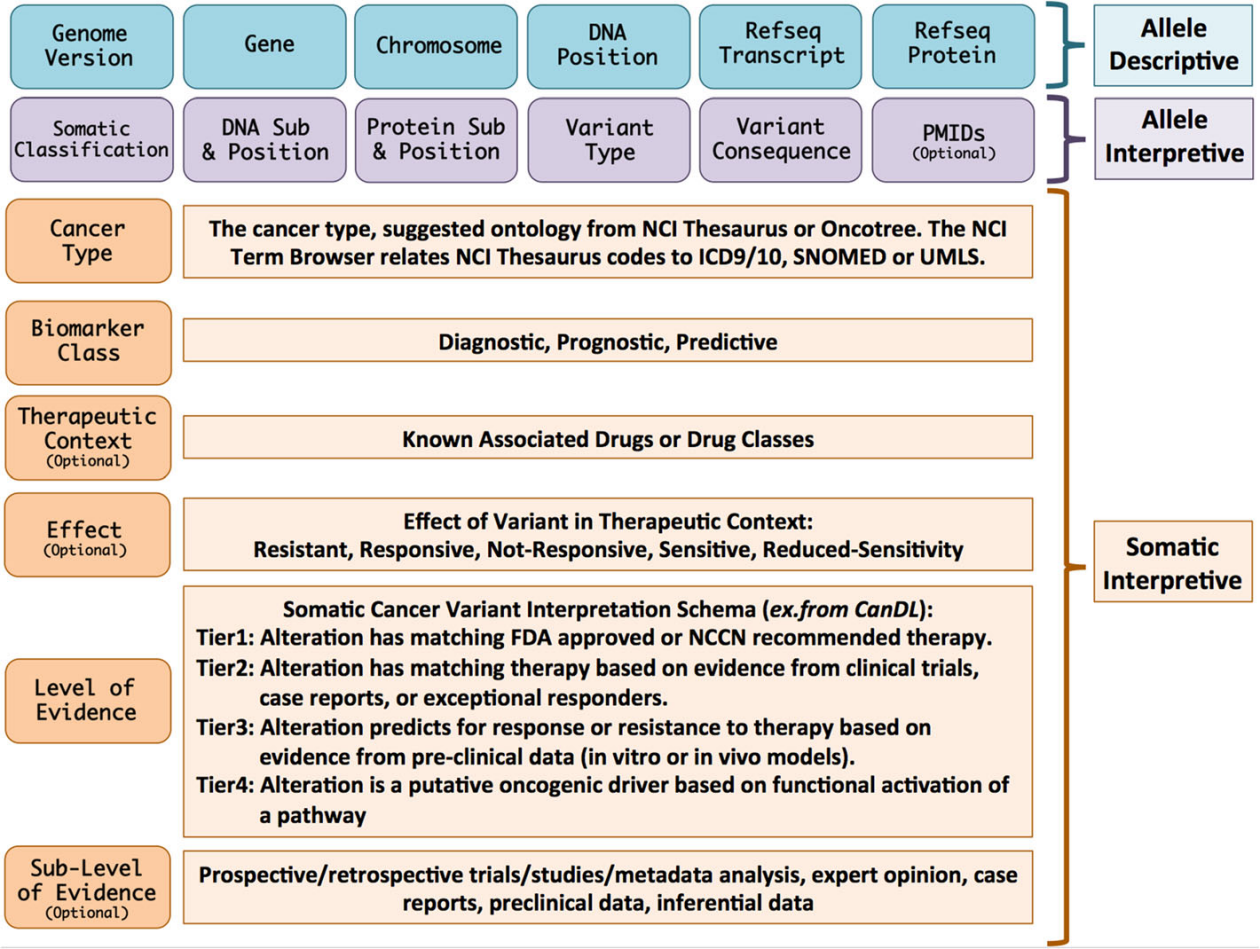
Minimum variant level data (MVLD) for somatic variant curation. The top two levels (*blue* and *purple*) contain fields generally in common use by most variant curation efforts, while the bottom set of fields (*orange*) are the cancer-interpretive fields. *ICD* International Classification of Diseases, *NCCN* National Comprehensive Cancer Network, *NCI* National Cancer Institute, *PMID* PubMed ID, *Sub* substitution, *SNOMED* Systematized Nomenclature of Medicine, *UMLS* Unified Medical Language System

Taking into account institutional surveys, group presentations and discussion as well as prior literature and current websites, we formed the consensus data elements of MVLD, as shown in Fig. 3 and described in further detail below. Throughout the descriptions, we suggest how these elements can be incorporated into the existing structure of ClinVar, although the intent of MVLD is to be a standard data structure not tailored to a specific database.

#### Allele descriptive fields

The first section of Fig. 3 (blue) incorporates overall standard fields already in common use to describe and characterize a genomic variant. *Genome Build* should be in GRCh37/GRCh38 format and should, if possible, use the actual version of the reference genome used to call the variant. *Gene Name* should be the Human Genome Organization (HUGO)-approved gene name. *Chromosome* and *DNA Position* should be the number or letter representation of the chromosome on which the variant is found and the corresponding genomic coordinate in HGVS format. For *Refseq Transcript* and *Refseq Protein* RefSeq transcripts and protein identifiers should be used. Since the transcript is often not known in sequencing data, all applicable transcripts may be used, or the most commonly accepted transcript may be used.

#### Allele interpretive fields

The second section of Fig. 3 (purple) pertains to the allele interpretive fields. Other than *Somatic Classification*, this section also contains generally standardized fields used by most curation efforts. The *Somatic Classification* field is necessary for cancer variant curation. Many centers do not yet require paired or matched normal sample sequencing, which is needed to distinguish cancer-specific variants from individual or rare germline variants. Suggested terms for the somatic classification are “Confirmed somatic”, “Confirmed germline”, or “Unknown.” The “Unknown” term could be a placeholder for submitters lacking matched normal sequencing or entities submitting data on behalf of literature or websites where paired sample information is not available. *DNA Substitution and Position* and *Protein Substitution and Position* should be written in HGVS format for both DNA and protein positions (if applicable). If there is a noncoding variant, the DNA position only may be supplied. For all other variant types, it is strongly suggested to include both DNA and protein annotations, but only one is required. *Variant Type* should represent the type of variant, such as single nucleotide variant (SNV), multi-nucleotide variant (MNV), insertion (INS), or deletion (DEL). Complex variants, such as deletion plus substitution, should be described as MNV. *Variant Consequence* should be the “molecular consequence” of a variant and rendered in the suggested terms “Nonsense”, “Missense”, “Silent”, “Frame shift”, “In-frame”, “3UTR”, “5UTR”, “Splice”, “Splice-region”, “Intronic”, “Upstream”, or “Downstream”. For *Variant Type* and *Variant Consequence*, these terms are all available in MISO Sequence Ontology codes as well [23]. *PubMed IDs* (PMIDs) are strings that reference supporting publications for the variant deposited. It is strongly suggested to use PMIDs to support variant evidence, but PMIDs are optional in this iteration of MVLD.

#### Cancer interpretive fields

The additional cancer-relevant fields are the main consensus data fields developed for the curation and dissemination of clinically relevant cancer variants. For *Cancer Type*, incorporating a standardized terminology for cancers when reporting variants is critically important to sharing datasets. Thus, a versatile ontology should be used to describe the cancer type. In clinical practice, sequencing requisition forms often require International Classification of Diseases (ICD) codes. However, this terminology set was not expressly created for describing cancers [24]. Several cancer-focused ontologies are available, such as National Cancer Institute (NCI) Thesaurus [25], a set of encoded terms to describe cancers and tissue pathology, and Oncotree, a set of 519 tumor types in 32 tissues [26]. Oncotree includes the NCI Thesaurus code for each cancer type. Through the NCI Term Browser, the NCI Thesaurus codes can also be related to other ontologies, such as the aforementioned ICD, or others such as SNOMED and UMLS. We suggest use of either NCI Thesaurus or Oncotree. The NCI Thesaurus can additionally describe histopathological tissue changes associated with cancers, while Oncotree, though limited to cancers, is very useful as a short, readily interpretable initialism of the cancer type (e.g. RGNT is rosette-forming glioneuronal tumor). The *Biomarker Class* field describes the clinical utility of the variant, and we suggest three standardized terms: “Diagnostic”, “Prognostic”, or “Predictive”. These are terms already in common use and are drawn from concepts proposed by the Institute of Medicine 2012 Translational Omics Report [27]. The *Therapeutic Context* field includes drugs that are specific to the variant reported. This field should first be populated with any FDA or National Comprehensive Cancer Network (NCCN) recommended treatment, followed by relevant drugs from commonly used drug databanks, including The DrugBank [28]. This is an optional field, though clinical relevance of a variant is greatly enhanced by including information of the relevant therapeutic context. The *Effect* field will hold keywords describing the effect of the variant in the therapeutic context. Deinstmann et al. [10] proposed a five-term vocabulary that we reason describes most cases: resistant, responsive, not-responsive, sensitive, reduced sensitivity. We have adopted this and added one field, “other”, to allow a free-text field descriptor only if none of the five descriptors apply. As this field is dependent upon the *Therapeutic Context*, it is also an optional field in this iteration of MVLD. While the *Level of Evidence* field can hold any variant-scoring framework, we suggest users adopt the forthcoming somatic variant interpretation guidelines issued by AMP. However, adopting a new framework may not be feasible for current projects or prior publications. Thus, any well-described interpretive or scoring framework may substitute here. The ClinVar field “Review Status (Assertion Method)” takes a similar approach. The user can submit a variant interpreted with a described published schema and the variant receives expert review with consideration of the referenced schema. One simple stratified somatic interpretation framework already in use is that of CanDL, the Cancer Driver Log [1]. In this somatic variant interpretation system, the level of evidence can be broken into four tiers, each tier using a short structured sentence to describe the utility of a cancer variant in clinical practice as follows: tier 1, “Alteration has matching FDA approved or NCCN recommended therapy”; tier 2, “Alteration has matching therapy based on evidence from clinical trials, case reports, or exceptional responders”; tier 3, “Alteration predicts for response or resistance to therapy based on evidence from pre-clinical data (in vitro or in vivo models)”; tier 4, “Alteration is a putative oncogenic driver based on functional activation of a pathway”. The *Sub-Level of Evidence* field further substantiates the *Level of Evidence* assertion and is generally composed of six possible subsections: (1) prospective trials/studies, (2) retrospective trials/studies and metadata analysis, (3) expert opinion, (4) case reports, (5) published preclinical data, and (6) inferential data or publications. Standardizing language and terminology in this field is important and will be an ongoing effort as MVLD further develops. In this iteration of MVLD, this field is optional. For prospective and retrospective trials and studies and metadata analysis, we recommend supplying the clinical trial number (NTC) for any clinical trial, which can be found on websites such as the NIH repository for clinical trials (ClinicalTrials.gov), MolecularMatch, or the International Clinical Trials Registry*.* For case reports or published preclinical data, always cite at least the PMID. Case reports are a single, unique observation in an individual (for example, [29]). Pre-clinical data are often functional data that have not been tested in a clinical trial but have strong implications for clinical utility (for example, [30]). For expert opinion, cite the date, name, and academic or medical affiliation of the expert or members of an expert panel. For published inferential data (for example, [31]), cite the PMID, or for in silico predictions, cite the name of the programs used.

The Somatic WG is implementing MVLD with ClinVar on a dataset from Baylor College of Medicine Advancing Sequencing Into Childhood Cancer Care (BASIC^3^) through the NIH-funded CSER. This set of somatic variants derives from pediatric solid tumor sequencing [7]. The published dataset was first transformed into a standard ClinVar submission to understand how current fields in ClinVar may be adapted to refine somatic variant handling. Then, the data were transformed to MVLD fields and categories such as “Biomarker Class”, which were not initially included in the dataset reviewed by CSER working group members. Currently we are seeking other datasets and groups who would like to upload somatic variants to ClinVar or test the MVLD format. We provide an example of MVLD in using the BASIC^3^ dataset in Additional file 1 (“Example of MVLD data format”). The completed, published BASIC^3^ dataset in MVLD format, with relation of the MVLD fields to those in ClinVar, will be made available as an example file for groups interested in testing MVLD formatting on their data.

## Discussion and conclusion

In its current state, the relevant information for the clinical use of a cancer variant is often dispersed, with different formats for similar information or relevant information missing. Inconsistency in cancer variant data creates knowledge gaps, complicates the exchange of cancer variant data, and uses considerable resources for repeated data transformations. In creating MVLD through a consensus approach, and promoting the adoption of a standardized framework across stakeholders in cancer variant curation, the Somatic WG of ClinGen aims to minimize redundant data handling and create a consistent set of elements for the clinical utility of cancer variants.

For a set of data elements such as MVLD to become a standard relies heavily on user/community adoption, uptake, and continued usage. The membership in ClinGen Somatic WG spans multiple institutions and includes at least five major cancer variant curation knowledge bases as well as multiple representatives from industry. In addition, members of the ClinGen Somatic WG are collectively members of many current major efforts to curate the cancer genome, including CSER, GA4GH, GENIE, Oncology Research Information Exchange Network (ORIEN), and The Cancer Genome Atlas (TCGA). MVLD was developed through a consensus approach with input from multiple groups that agree on the necessity of a practical and useful data standard for cancer variant curation. We are also working closely in partnership with ClinVar both to understand somatic variant handling and to test migration from MVLD to ClinVar submissions. MVLD will likely evolve during a testing and adoption phase, and we are actively reaching out to groups interested in submitting somatic data to ClinVar to assist with data handling, increase somatic submissions to ClinVar, and solicit MVLD testing and feedback. In subsequent iterations of MVLD, we plan to extend the reach to international databases such as COSMIC. Also, creating a parallel somatic rating schema to that of the current germline variant “star” system is currently being discussed, as are the creation of expert panels for somatic variant reviews.

It is important to note that in its current format, MVLD mainly applies to aggregated variant-level data and not necessarily to case-level data. For example, a recent study describes an individual with four somatic variants in a rosette-forming glioneuronal tumor (RGNT) and a pathogenic germline variant [32]. At the variant level, MVLD does not capture information relating multiple variants in one sample. This is important to consider because individual samples can often have multiple somatic variants and possible drug contraindications [14]. Currently, initiatives such as GA4GH, GENIE, and ORIEN are focusing efforts on modeling case-level data. In addition, the MVLD currently requires Refseq identifiers, as well as select ontologies that can be mapped to NCI Thesaurus. Although total transcript content may be comparatively increased in sets such as Gencode, UCSC, or Ensembl, the true impact on limitation for variants in MVLD format is currently unknown. We suggest that using ClinVar to track bulk somatic variant submissions lacking mappable Refseq identifiers could help gauge estimations. Also, additional ontologies may be rapidly added. For example, Disease Ontology also maps to NCI codes and thus further iterations may offer a “local ontology” optional field alongside a more circumspect selection of required standardized terms [33]. While standardization often necessitates limitation, current tools in development to convert between ontologies and annotation sets will greatly enhance the perspective usage scope. In addition, MVLD captures only DNA sequencing data and does not in the current format capture RNA data, structural variation data, “outcomes” level data, or other cancer-relevant test data. However, additional types of data beyond next-generation sequencing variants will build a more personalized approach to cancer care, and handling the complex challenge of standardizing diverse data is made simpler by having first a basic set of common data elements for cancer variant curation.

Standardizing the data elements that represent a somatic variant is critically important to enhancing communication and utility of genetic data for clinicians, researchers, and the public. The FDA has recently proposed creating an FDA recommendation for variant databases, and standardization of data across databases is a core feature of their proposed initiative. A standard set of key data elements specified using controlled vocabularies to describe somatic variants from clinical tests will enable large-scale analysis of molecular diagnostic and theranostic data from multiple sources and drive forward cancer precision medicine, as well as ensure continued and broad use of clinical and research data.

## Additional file

Additional file 1: Example of the MVLD data format. (XLSX 32 kb)

## Abbreviations

AMP: Association for Molecular Pathology
BASIC^3^: Baylor College of Medicine Advancing Sequencing Into Childhood Cancer Care
CanDL: Cancer Driver Log
CIViC: Clinical Interpretations of Variations in Cancer
ClinGen: Clinical Genome Resource
COSMIC: Catalog of Somatic Mutations in Cancer
CSER: Clinical Sequencing Exploratory Research
FDA: Food and Drug Association
GA4GH: Global Alliance For Genomics and Health
GENIE: Genomics, Evidence, Neoplasia, Information, Exchange
ICD: International Classification of Diseases
MNV: Multi-nucleotide variant
MVLD: Minimum Variant Level Data
NCCN: National Comprehensive Cancer Network
NCI: National Cancer Institute
NIH: National Institutes of Health
ORIEN: Oncology Research Information Exchange Network
PMID: PubMed ID
WG: Working Group
